# Atypical Presentation of Pneumocephalus Post Ventriculoperitoneal Shunt in a Patient With a History of Endoscopic Endonasal Skull Base Approach: A Case Report

**DOI:** 10.7759/cureus.78123

**Published:** 2025-01-28

**Authors:** Rahaf M Alalawi, Waseem Th. Alharthi, Reem F Almaimani, Abdulaziz A Almusa

**Affiliations:** 1 College of Medicine, Taif University, Taif, SAU; 2 College of Medicine, Batterjee Medical College for Science and Technology, Jeddah, SAU; 3 Neurosurgery, Prince Sultan Military Medical Hospital, Riyadh, SAU

**Keywords:** csf leak, endoscopic endonasal, epidermoid cyst, hydrocephalous, tension pneumocephalus

## Abstract

Pneumocephalus, commonly seen after trauma, surgical intervention, or meningitis, is rarely associated with ventriculoperitoneal shunt (VPS) procedures. We present a unique case of tension pneumocephalus in a 26-year-old female who experienced two distinct episodes of pneumocephalus. She presented with right-sided facial numbness, hearing loss, blurry vision, and gait disturbance. Magnetic resonance imaging (MRI) revealed a large extra-axial lesion at the right petrous apex extending to the middle cranial fossa. The patient underwent an extended endoscopic endonasal approach for tumor resection, and the pathological diagnosis revealed an epidermoid cyst. Postoperatively, she improved. However, she developed abducens nerve palsy followed by extensive pneumocephalus with intraventricular extension, necessitating skull base defect repair. Six weeks later, she presented with acute hydrocephalus secondary to meningitis from *Klebsiella pneumoniae*, confirmed by positive cerebrospinal fluid (CSF) cultures. She was treated with external ventricular drainage and antibiotic therapy, after which a VPS was inserted. Three days post-shunt insertion, the patient developed left-sided hemiparesis and swallowing dysfunction due to localized pneumocephalus within the tumor cavity compressing the brainstem. Following additional surgical intervention, her hemiparesis and other symptoms resolved. This case highlights the potential for tension pneumocephalus following ventriculoperitoneal shunt insertion for hydrocephalus. The siphon effects of CSF shunting can cause excessively negative intracranial pressure. Combined with a postoperative skull base defect, this can lead to air ingress through the defect (ball valve mechanism), causing pneumocephalus.

## Introduction

Pneumocephalus, also known as intracerebral aerocele or pneumatocele, refers to the accumulation of air within the cranial cavity [1]. Developing due to a connection between the atmosphere and the intracranial space, it was first documented by Lecat et al. in 1741. Approximately 74% of cases are linked to head trauma [2]. Neurosurgical procedures, craniofacial trauma, endoscopic sinus surgery, skull base tumors, frontal sinus cranialization, infections, and diagnostic or neurosurgical interventions like pneumoencephalography or lumbar puncture are additional causes; however, spontaneous occurrences are rare [2,3].

Pneumocephalus may be classified based on anatomical localization such as extradural, subdural, subarachnoidal, intraparenchymal, or intraventricular [3]. The pathomechanism involves two key factors: a reduction in intracranial pressure and concurrent defects in the dura. These factors can be attributed to either a ball-valve mechanism, causing a rapid air volume increase in the cranial cavity without compensatory cerebrospinal fluid (CSF) outflow, or CSF leakage leading to intracranial hypotension and subsequent air aspiration [3]. Considered a serious complication, pneumocephalus may necessitate neurosurgical intervention, especially in cases associated with clinical deterioration. There is an increased risk of developing meningitis in the presence of pneumocephalus.

The clinical presentation of pneumocephalus encompasses symptoms such as headache, nausea, vomiting, dizziness, seizures, recurrent meningitis, and alterations in consciousness. In rare instances, eye pain and diplopia resulting from cranial nerve palsy have been reported [2]. Conservative approaches to treating pneumocephalus involve bed rest, oxygen therapy, prophylactic antibiotic administration, avoidance of positive pressure, and pain management. Surgical interventions include repairing bony defects through endoscopic endonasal surgery, osteoplastic flap surgery of the frontal sinus using a bicoronal incision, and craniotomy. The choice of treatment is influenced by the patient's clinical condition and age [2].

Here, we present a case of tension pneumocephalus following ventriculoperitoneal insertion in a patient with an existing skull-base defect.

## Case presentation

**Figure 1:**
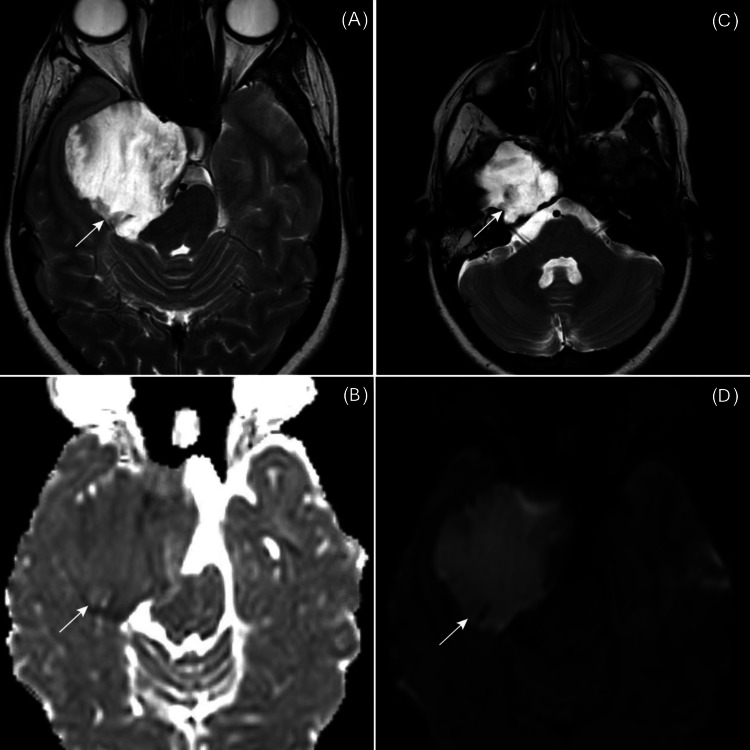
Axial MRI and diffusion-weighted imaging (DWI) of a hyperintense extra-axial lesion at the right petrous apex (A) T2-weighted MRI shows a hyperintense lesion at the right petrous apex with a mass effect on surrounding structures. (B) The ADC (apparent diffusion coefficient) map shows restricted diffusion within the lesion, indicative of high cellularity. (C) T2-weighted MRI highlights the lesion’s extension into the middle cranial fossa, further compressing adjacent areas. (D) DWI confirms restricted diffusion within the lesion, supporting the diagnosis of a tumor.

A 26-year-old female was referred to our center from a peripheral hospital. She presented with right facial numbness, intermittent headache, blurry vision, decreased hearing, and gait disturbance. Her numbness was more pronounced in the V2 and V3 distributions and was present throughout the day, without any specific aggravating or relieving factors. She had a history of a ruptured tympanic membrane three years ago, which had healed by the time of her initial presentation. She reported no other significant medical or surgical history. Magnetic resonance imaging revealed a large, hyperintense extra-axial lesion at the right petrous apex on T2-weighted images, with diffusion restriction on diffusion-weighted images. The lesion extended into the middle cranial fossa, causing a mass effect with displacement of the adjacent brainstem (Figure 1).

The patient underwent an extended endoscopic endonasal approach for tumor resection. During the surgery, the dura mater was not exposed and no dural lesions were noted. The procedure was completed without an overt intraoperative CSF leak. The immediate frozen section diagnosis of the specimen revealed keratin flakes with blood, consistent with an epidermoid cyst. Postoperatively, the patient showed improvement in facial numbness and gait but developed abducens nerve palsy.

**Figure 2:**
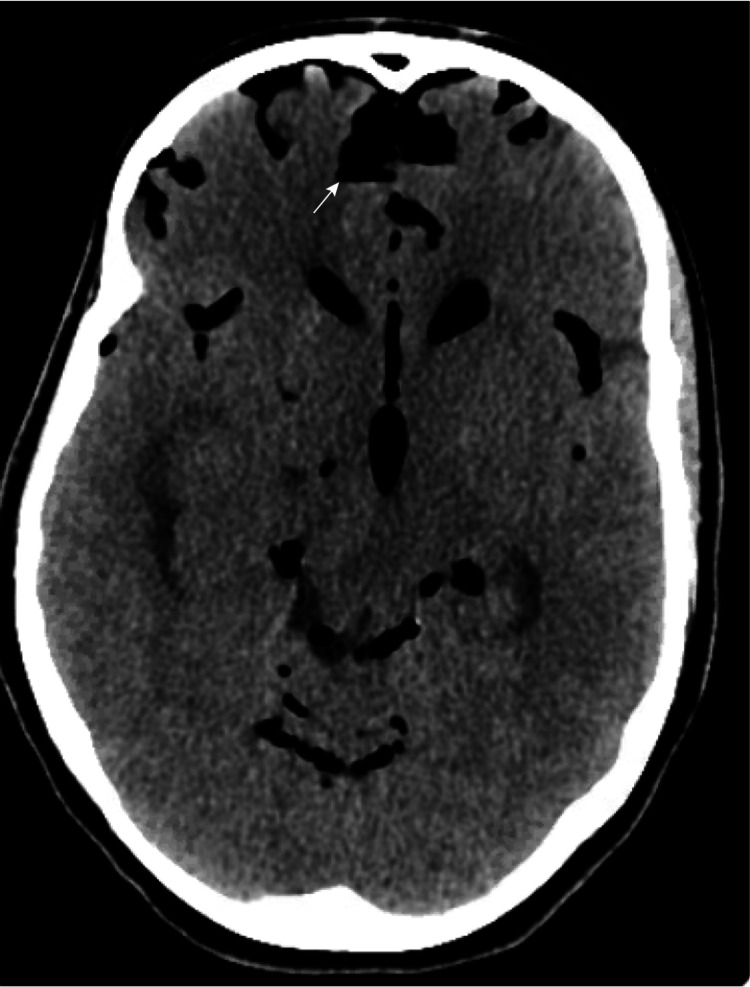
CT brain indicating extensive pneumocephalus five days after initial tumor resection

Five days later, the patient developed a sudden severe headache. A plain CT brain revealed extensive pneumocephalus (Figure 2).

Endoscopic endonasal reconstruction with fat graft was performed. A skullbase defect with evident cerebrospinal fluid leak was identified and copious irrigation was done within the cavity. Air bubbles were noticed coming out and the Valsalva maneuver was done after reconstruction with no leak. She was discharged home three days later in good condition.

Six weeks later, the patient was presented to her original peripheral hospital with low-grade fever, headache, blurry vision, and three episodes of seizures. A diagnosis of acute hydrocephalus secondary to meningitis was made and supported by a positive culture for *Klebsiella (K.) pneumoniae* in a CSF sample obtained intraoperatively after the insertion of right frontal external ventricular drainage. Subsequently, the patient was treated with 2g of cefepime, administered intravenously every 6 hours for 14 days, for a *K. pneumoniae* infection. After the clearance of CSF, a ventriculoperitoneal shunt was inserted. The shunt system used was a programmable valve, initially set to a medium-pressure setting.

**Figure 3:**
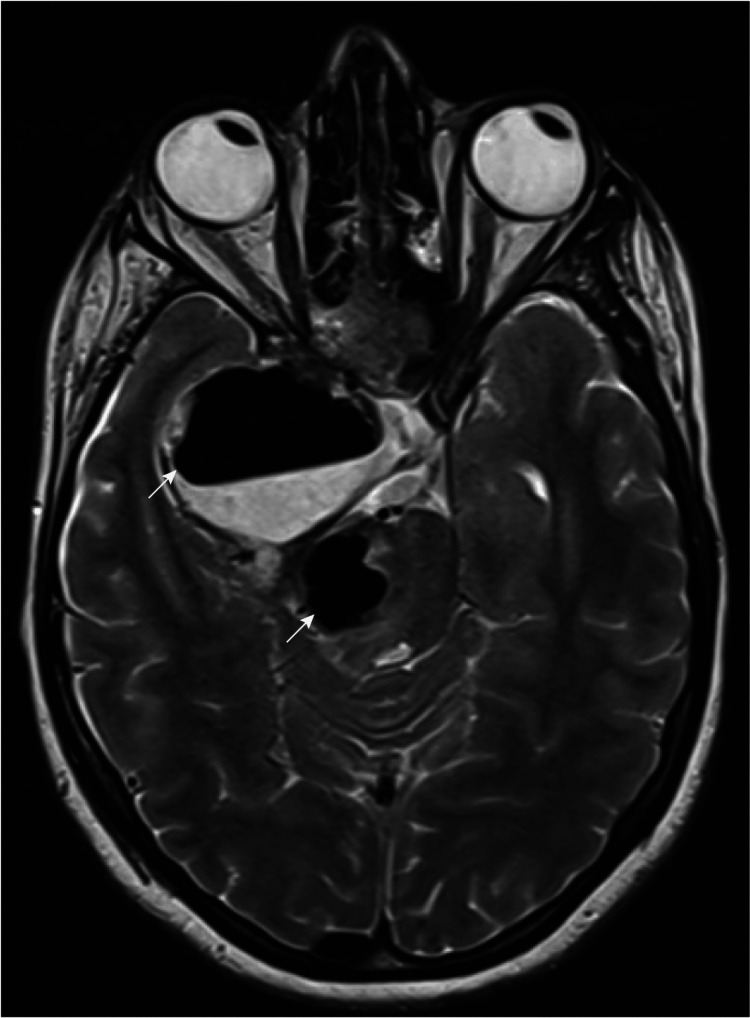
MRI imaging demonstrating localized pneumocephalus within the tumor cavity

**Figure 4:**
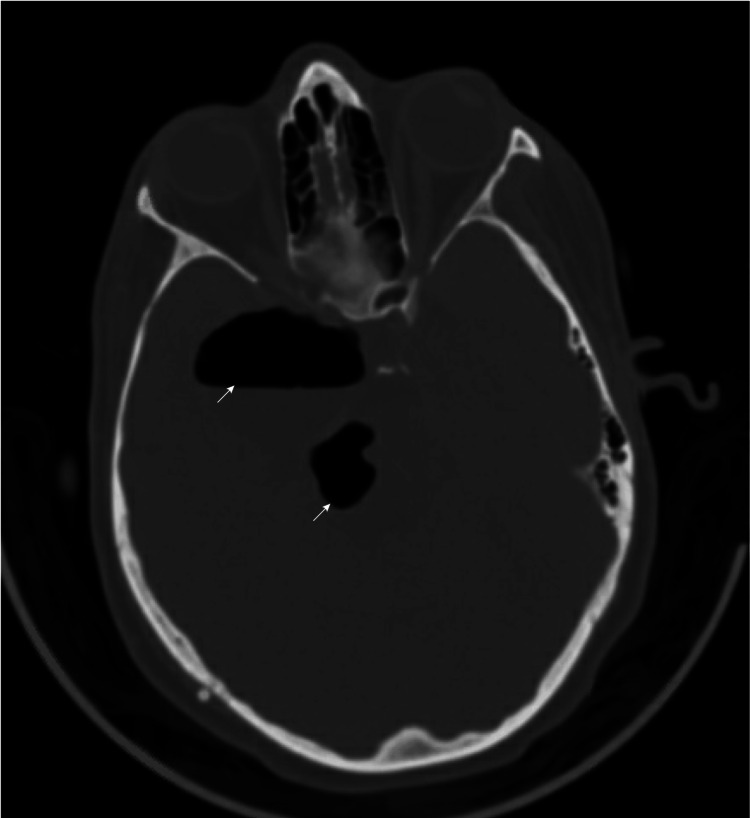
CT imaging demonstrating localized pneumocephalus within the tumor cavity

Three days later, the patient developed a left-side hemiparesis with swallowing dysfunction. Plain CT brain and T2 MRI scans show localized pneumocephalus within the tumor cavity, compressing the brainstem (Figure 3 and Figure 4).

**Figure 5:**
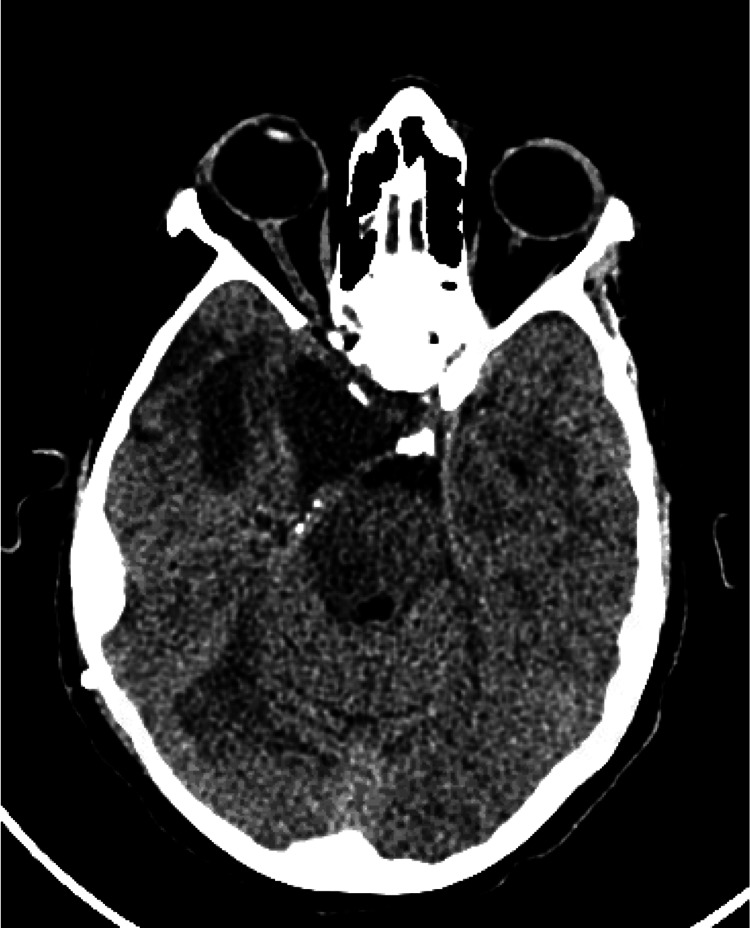
Plain CT brain post-repair, demonstrating resolution of pneumocephalus following endoscopic repair

Another endoscopic skull base defect repair was performed, during which the defect was identified and sealed with an additional fat graft, as the fat from the prior surgery was not reused. Extensive irrigation was carried out within the cavity, and a Valsalva maneuver confirmed the absence of further CSF leakage. As a result, the patient showed improved pneumocephalus with resolution of hemiparesis and was transferred to a general ward, followed by an extensive rehabilitation program. She was discharged home a few weeks later and was able to ambulate independently with complete resolution of her initial symptoms. Abducent nerve palsy improved completely three months postoperatively (Figure 5). The patient was followed up for a total of six months, during which she showed complete recovery without any recurrence of infection or other complications.

## Discussion

Trauma accounts for approximately 75% of pneumocephalus cases; however, it can also occur as a complication of routine neurological interventions such as the insertion of a ventriculoperitoneal (VP) shunt [4].

The presented case of pneumocephalus illustrates an uncommon clinical scenario. The patient's initial presentation included symptoms such as right facial numbness, intermittent headaches, blurry vision, and gait disturbances, coupled with a history of a ruptured tympanic membrane, which set the stage for a complex diagnostic journey. Scattered case reports have described instances of tension pneumocephalus following endoscopic skull base surgery or VP shunt implantation [4]. While most patients experience common symptoms of elevated intracranial pressure (ICP), such as headache, nausea, and vomiting, more severe cases may present with altered mental status, seizures, focal neurological impairments, and even death [5].

After the identification of a massive right middle cranial fossa mass on unenhanced CT imaging, concerns regarding mass effect and displacement of the neighboring brainstem arose. Following the decision to perform endoscopic extended endonasal transsphenoidal tumor resection, the patient experienced an unforeseen postoperative complication manifested as an acute onset of severe headache.

In such cases, a cerebrospinal fluid (CSF) fistula should be considered, and imaging scans can assist in the diagnosis, often revealing a significant volume of intracranial air. High-resolution (HR) CT and magnetic resonance imaging (MRI) cisternography are the primary modalities for investigating CSF leakage [6]. Non-contrast head CT is considered the gold standard for diagnosing pneumocephalus due to its excellent specificity, speed, and ease of access, being able to detect as little as 0.55 mL of cerebral air due to its -1000 Hounsfield coefficient [7].

Once identified, tension pneumocephalus necessitates immediate neurosurgical examination and intervention to prevent lasting brain damage, specifically by releasing the trapped air [8]. In our case, endoscopic endonasal repair with a fat graft was performed, where a skull base defect with an obvious CSF leak was discovered and managed. The patient was discharged three days later.

However, six weeks later, the patient presented to a peripheral hospital with subsequent complications, including acute hydrocephalus secondary to meningitis, necessitating antibiotic therapy and VP shunt insertion. 

Several theories explain the mechanisms behind pneumocephalus. One notable theory involves a decrease in ICP due to CSF loss, which can occur artificially through a ventriculoperitoneal shunt. This loss of CSF results in intracranial hypotension, creating a vacuum effect that paradoxically draws atmospheric air into the cranial cavity. This situation persists until there is a balance between the pressures inside and outside the skull. This phenomenon is referred to as the "inverted soda bottle effect" of Horowitz and Lunsford [7].

In this case, three days post-surgery, the patient developed left-sided hemiparesis and swallowing dysfunction. A plain CT scan and T2 MRI images revealed localized pneumocephalus within the tumor cavity, compressing the brainstem. Tension pneumocephalus was suspected based on non-specific but significant symptoms, including headache, decreased level of consciousness, cranial nerve palsy, and hemiparesis [9].

The patient was managed with another endoscopic endonasal repair. Thus, early and targeted interventions, along with therapy and follow-up imaging, indicated a positive trend with improved pneumocephalus and neurological function. Pneumocephalus should be evaluated as a potential diagnosis, particularly in cases with a history of relevant predisposing factors [10]. This case study highlights the importance of vigilance, prompt recognition, and multidisciplinary management, which are essential for effectively improving patient outcomes in the context of this rare and often overlooked condition.

## Conclusions

This case highlights the complexity and potential severity of pneumocephalus, particularly in scenarios lacking overt trauma or immediate neurosurgical intervention. Surgical management, including endoscopic endonasal reconstruction, was essential in resolving the pneumocephalus and improving neurological function. This case emphasizes the importance of considering pneumocephalus in the differential diagnosis, particularly in patients with a history of predisposing factors such as tympanic membrane rupture and intracranial surgery. It also highlights the necessity for multidisciplinary management involving neurosurgery, infectious disease, and critical care teams to optimize patient outcomes.
